# Cryopreservation of human spermatozoa by freezing testicular seminiferous tubule with novel cryopiece

**DOI:** 10.1111/and.14356

**Published:** 2021-12-26

**Authors:** Feng Lin, Beihong Zheng

**Affiliations:** ^1^ Center of Reproductive Medicine Fujian Maternity and Child Health Hospital Affiliated Hospital of Fujian Medical University Fuzhou China

**Keywords:** cryopreservation, freeze–thaw sperm quality, human testicular seminiferous tubule, novel cryopiece, testicular cell suspensions

## Abstract

This study was carried out to evaluate the effectiveness of cryopreservation of human spermatozoa by freezing testicular seminiferous tubule with a new cryocarrier named ‘novel cryopiece’. Testicular tissue (TT) was collected from patients who underwent diagnostic testicular biopsy. Overall, 35 TT samples were obtained. Each TT sample was equally divided into four groups named (e.g. G1, G2, G3 and Gc). G1 was frozen as testicular seminiferous tubule using novel cryopiece, G2 was frozen as testicular cell suspensions using novel cryopiece, G3 was frozen as testicular cell suspensions using 0.25 ml straw, and Gc was not frozen. The samples in G1 and G2 experimental groups were cryopreserved in five separate aliquots and stored in the same cryovial. The freeze–thaw sperm DNA fragmentation index (DFI) of G2 was lower than that of G3 (20.27 ± 5.40 vs 23.55 ± 6.02; *p *= 0.004). After thawing, spermatozoa could be found in all 35 testicular seminiferous tubule specimens in G1; however, it could not be found in 2 of 35 (5.7%) and 1 of 35 (2.9%) testicular cell suspensions samples in G2 and G3 respectively. This study indicates that novel cryopiece presented for the cryopreservation of testicular seminiferous tubules and testicular cell suspension is simple and effective.

## 1 INTRODUCTION

The amount of spermatozoa recovered by techniques such as testicular sperm extraction (TESE) (Erdem et al., 2017) in patients with obstructive azoospermia (OA) is very limited. The testicular spermatozoa injected into oocytes by the intracytoplasmic sperm injection (ICSI) technique form embryos that are transferred into the uterus to obtain a clinical pregnancy. The success of TESE and ICSI in obtaining fertility in patients has been known for a long time (An et al., 2018).

Although the amount of spermatozoa obtained by testicular biopsy is small, the retrieved spermatozoon is a valuable resource for patients with azoospermia (Desai et al., 2004). Indeed, the success of assisted reproductive therapy is often unpredictable, failure implies the need for uncomfortable and painful repeated testicular biopsies, and this can also increase the risk of orchitis, disruption of the blood–testis barrier, and production of antisperm antibodies (Schlegel & Su, 1997). Cryopreservation of testicular spermatozoa allows patients to perform multiple treatments of assisted reproduction with a small amount of testicular spermatozoa (Liu & Li, 2020). Therefore, in order to avoid damage to the testis caused by repeated surgery, there is the need to freeze testicular spermatozoa found in the first testicular biopsy.

Because the DNA of testicular spermatozoa is not stable enough and the cell membrane is not completely mature (Küpker et al., 2002), the recovery rate of testicular sperm cryopreserved by conventional semen freezing method is only 1% (Borini et al., 2000) and is not satisfactory.

To date, several sperm cryocarriers have been developed, including human or hamster empty zonae (Desai et al., 2004; Walmsley et al., 1998), micro‐straw (Huang et al., 2020; Isachenko et al., 2005), ICSI needle (Sohn et al., 2003), Cryotop (Chen et al., 2015), Cellsleeper (Endo et al., 2012) and Cryopiece (Sun et al., 2017). However, the efficacy of these cryocarriers is suboptimal; therefore, there is the need for the development of novel cryopreservation carriers specifically designed for small numbers of spermatozoa (AbdelHafez et al., 2009; Liu & Li, 2020).

In the present article, we novelly use the cryopreservation of testicular spermatozoa by freezing testicular seminiferous tubule. Additionally, we developed a new carrier, named novel cryopiece. In order to verify the effectiveness of novel cryopiece, we measured the motility and survival rate, as well as the DNA fragmentation index (DFI) of testicular spermatozoa before and after freezing.

## 2 METHODS

### 2.1 Design of novel cryopiece

**FIGURE 1:**
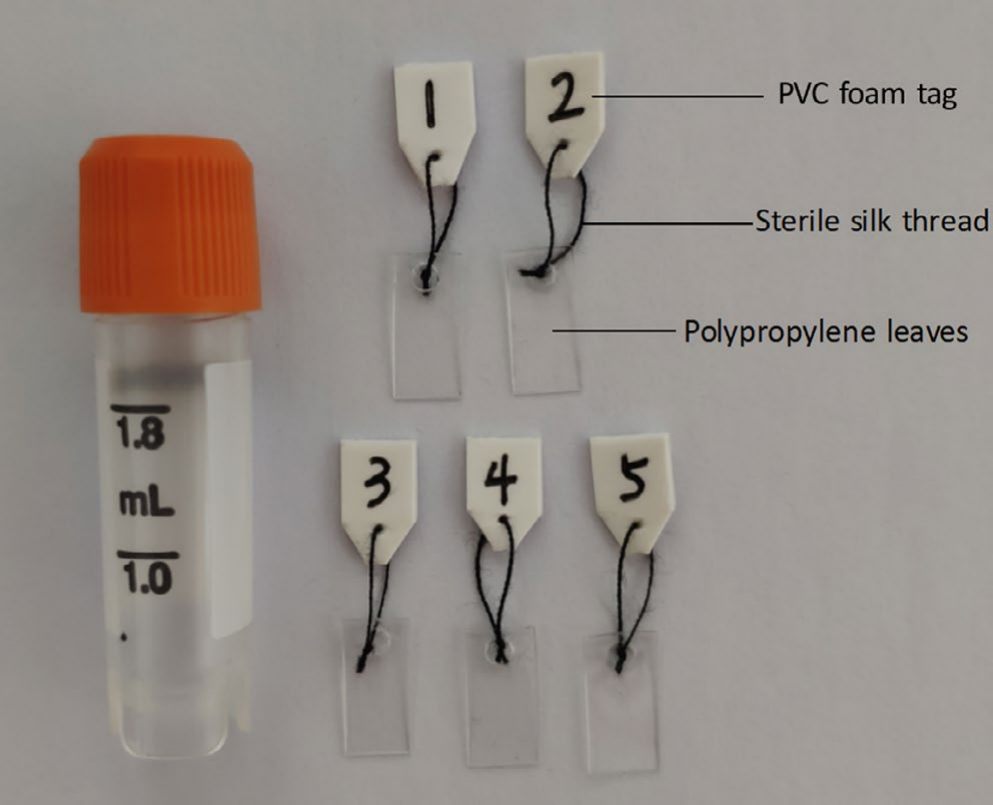
Novel cryopiece consists of an outer cannula and five non‐toxic polypropylene leaves

**FIGURE 2:**
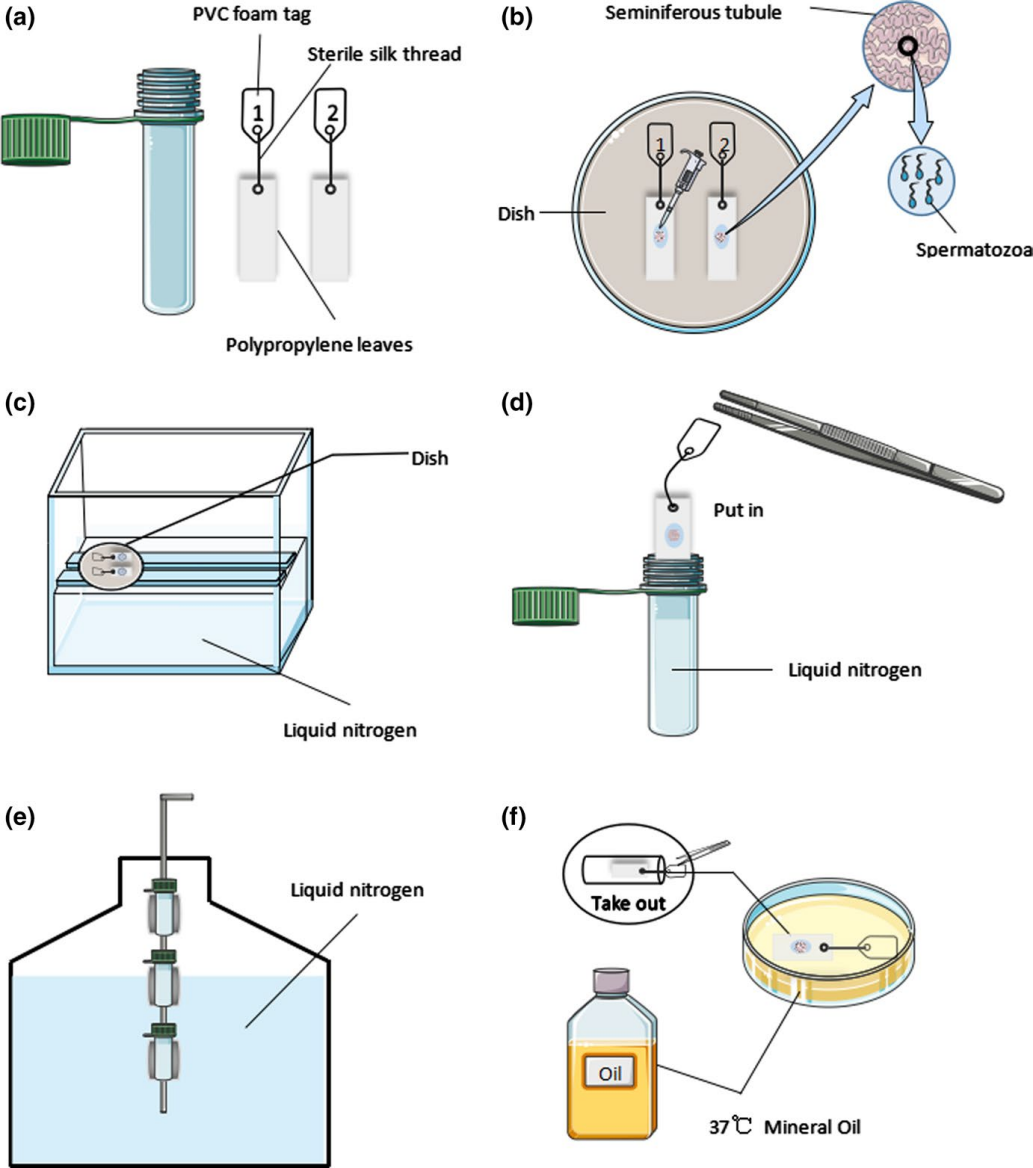
Schematic representation of the novel cryopiece freezing–thawing procedure. (a) Prepare a 1.8 ml cryotube and five polypropylene leaves attached with foam tags. (b) Polypropylene leaves were placed on a dish. 20 μl of Sperm Cryoprotectant (origio) was dropped on each of the five non‐toxic polypropylene leaves; then, the testicular seminiferous tubule was equally divided into five parts, each of which was put in a drop of Sperm Cryoprotectant. (c) The dish with the polypropylene leaves was exposed to liquid nitrogen vapour for 10 minutes. (d) Then, the polypropylene leaves were plunged into a 1.8ml cryovial filled with liquid nitrogen. (e) Cryovial was stored in liquid nitrogen for at least 1 week. (f) Polypropylene leaves loaded with seminiferous tubule were removed from liquid nitrogen and immediately inserted into ICSI dishes covered with mineral oil at 37℃ for 5–10 min

Novel cryopiece consists of an outer cannula and five non‐toxic polypropylene leaves (Figures 1 and 2a), which could be manufactured independently by the embryo laboratory. The outer cannula is a 1.8 ml conventional sperm cryovial (ThermoFisher Scientific). The non‐toxic polypropylene leaf is 1.0 cm long and 0.5 cm wide and is attached with a foam tag made from non‐toxic polyvinyl chloride (PVC) foam board using a sterile silk thread (ETHICON MERSILK SA82G). Before novel cryopiece fabrication, the polypropylene leaf must be sterilized by high‐pressure steam sterilization method (pressure 147 kpa, steam temperature 121°C 20 min) and the PVC foam tag was washed with sterile water for injection. Multiple polypropylene leaves can be stored simultaneously in one cryovial.

### 2.2 Sample preparation

#### 2.2.1 Acquisition of testicular tissue

Our prospective cohort study of testicular sperm cryopreservation included patients who underwent testicular fine needle aspiration (TFNA) (Foresta et al., 1992) for diagnostic testicular biopsy in Fujian Provincial Maternity and Children's Hospital from 1 December 2018 to 28 September 2020. The testicular puncture site was the area with relatively few arterial branches on the lateral and superior surface of the testis, and no significant testicular hematoma was observed after surgery in all patients who underwent TFNA. Overall, 40 testicular tissue (TT) samples were obtained from 35 patients with obstructive azoospermia (OA), four patients with non‐obstructive azoospermia (NOA) and one patient with hypospermatogenesis. 35 TT samples from OA patients were used for the successive experiments (Table 1). All patients signed an informed consent form before surgery for the use of their testicular tissue samples for scientific research purposes, and testicular biopsy procedures were performed by the same physician.

**TABLE 1:** Characteristics of included patients.

Patient	Age (y)	Volume of Left and right testes (ml)	FSH (mIU/mL)	AZF gene	Chromosome	Diagnosis	Aetiology
1	29	20;18	2.12	Normal	46,XY	OA	Seminal duct obstruction
2	37	15;18	6.77	Normal	46,XY	OA	Seminal duct obstruction
3	31	20;15	5.2	Normal	46,X,t(Y;22)(p11.2;q11.2)	NOA	Chromosomal abnormalities
4	28	12;12	12.27	Normal	46,XY	NOA	Seminal duct obstruction
5	33	18;15	4.4	Normal	46,XY	OA	Seminal duct obstruction
6	22	18;22	6.52	Normal	46,XY	OA	Seminal duct obstruction
7	24	15;20	2.98	Normal	46,XY	OA	Seminal duct obstruction
8	32	16;15	7	Normal	46,XY	OA	CBAVD
9	37	18;17	9.23	Normal	46,XY	OA	Seminal duct obstruction
10	51	18;18	10.13	Normal	46,XY	hypospermatogenesis	Man of advanced age
11	32	14;14	4.98	Normal	46,XY	OA	Diabetes Anejaculation (AE)
12	30	10;10	1.85	Normal	46,XY	NOA	Patient with spinal tumour after radiochemotherapy
13	35	18;15	7.98	Normal	46,XY	OA	CBAVD
14	25	16;18	5.87	Normal	46,XY	OA	Seminal duct obstruction
15	30	14;15	4.74	Normal	46,XY	OA	Seminal duct obstruction
16	34	22;20	3.99	Normal	46,XY	OA	Seminal duct obstruction
17	35	18;16	4.24	Normal	46,XY	OA	CBAVD
18	36	15;16	5.13	Normal	46,XY	OA	Diabetes Anejaculation (AE)
19	38	22;25	5.04	Normal	46,XY	OA	CBAVD
20	28	15;25	9.39	Normal	46,XY	OA	Anejaculation (AE)
21	32	10;20	6.26	Normal	46,XY	NOA	Left inguinal cryptorchidism
22	43	16;16	3.89	Normal	46,XY	OA	Seminal duct obstruction
23	29	22;18	4.52	Normal	46,XY	OA	Seminal duct obstruction
24	33	16;18	2.87	Normal	46,XY	OA	Seminal duct obstruction
25	26	14;15	5.34	Normal	46,XY	OA	CBAVD
26	28	16;15	3.23	Normal	46,XY	OA	Seminal duct obstruction
27	39	25;25	3.96	Normal	46,XY	OA	Seminal duct obstruction
28	31	15;18	4.69	Normal	46,XY	OA	Seminal duct obstruction
29	23	20;21	8.23	Normal	46,XY	OA	Seminal duct obstruction
30	26	19;20	1.78	Normal	46,XY	OA	Seminal duct obstruction
31	34	16;15	2.69	Normal	46,XY	OA	Seminal duct obstruction
32	34	10;20	4.43	Normal	46,XY	OA	Seminal duct obstruction
33	25	15;15	3.98	Normal	46,XY	OA	Seminal duct obstruction
34	22	20;20	5.67	Normal	46,XY	OA	CBAVD
35	26	20;15	7.67	Normal	46,XY	OA	Seminal duct obstruction
36	31	22;22	7.17	Normal	46,XY	OA	Seminal duct obstruction
37	34	18;18	3.89	Normal	46,XY	OA	Seminal duct obstruction
38	37	10;20	4.67	Normal	46,XY	OA	Seminal duct obstruction
39	30	12;15	2.54	Normal	46,XY	OA	CBAVD
40	29	20;15	6.43	Normal	46,XY	OA	Seminal duct obstruction

Note

The normal value of FSH was 2–8 mIU/mL.

AZF, azoospermia factor; CBAVD, congenital bilateral absence of the vas deferens; NOA, non‐obstructive azoospermia; OA, obstructive azoospermia.

This study was approved by the Ethics Review Committee of Fujian Maternity and Child Health Hospital, Affiliated Hospital of Fujian Medical University on 20 March 2018 (reference YCXM 2018‐037).

#### 2.2.2 Exclusion criteria

Patients with the following characteristics were excluded: FSH >2 times the upper limit of normal; AZF gene deletion; volume of both testes <6 ml; chromosome 47,XXY or 46,XX; poor living habits (long‐term smoking, drinking and drug abuse); high‐risk occupations (drivers, practitioners in high temperature environment, chemical industry practitioners and radiation exposure workers).

#### 2.2.3 Preparation of testicular seminiferous tubule

The total volume of the biopsied testicular tissue ranged from 4 to 25 mm^3^, due to the randomicity of the TFNA. The collected TT was put in a pre‐equilibrated sperm preparation medium (MediCult, Jyllinge, Denmark) at room temperature and sent to the laboratory for processing. The testicular tissue was sequentially transferred into 3 drops of 100 µl G‐GAMETE™ medium prepared in a Petri dish and thoroughly washed with two 1 ml syringe needles to remove as much blood and interstitial tissue as possible, leaving only the testicular seminiferous tubule. Each of 35 testicular seminiferous tubule samples was divided into two portions for freezing tests. One portion of the testicular seminiferous tubule samples was cryopreserved using cryopiece (as group 1, named G1), and the other portion was prepared as testicular cell suspensions.

#### 2.2.4 Preparation of testicular cell suspensions

Testicular seminiferous tubules were transferred into drops containing fresh sperm medium (G‐GAMETE™) in petri dishes and mechanically torn with two 1 ml syringe needles (Esteves & Varghese, 2012), which was then transferred into a centrifuge tube containing fresh sperm medium (G‐GAMETE™). The mixture was centrifuged at 500 G for 10 min. The supernatant was discarded. Then, the pellet was resuspended in 150 µl sperm medium and kept at 37℃ for at least 1 h. The testicular cell suspensions were divided into three aliquots: one was used to measure testicular sperm motility, survival rate and DFI before freezing (as control group, named Gc), and the remaining two aliquots were frozen with novel cryopiece and 0.25 ml straw respectively (as groups 2 and 3, named G2 and G3). For cryopreservation, the cryoprotectant solution (Origio, Denmark, REF 1067) was added to the testicular cell suspensions at a ratio of 1:1, and two groups of 100 µl cryoprotector‐sperm mixtures samples were equilibrated at room temperature for 15 min.

### 2.3 Freezing and thawing

#### 2.3.1 Testicular seminiferous tubule frozen with novel cryopiece

For freezing, seminiferous tubules of G1 were placed in the Sperm Cryoprotectant (Origio, Denmark) solution at room temperature for 15 min and then frozen in raw form. Polypropylene leaves were placed on a dish. 20 µl of Sperm Cryoprotectant (Origio, Denmark) was dropped on each of the five non‐toxic polypropylene leaves; then, the testicular seminiferous tubule was equally divided into five parts and transferred into Sperm Cryoprotectant (Origio, Denmark) droplets respectively. The volume of cryopreserved seminiferous tubules ranged from 0.4 to 2.5 mm^3^ (Figure 2b). The dish with the polypropylene leaves was exposed to liquid nitrogen vapour (4 cm above the level of the liquid nitrogen, −120℃) for 10 minutes (Figure 2c). Then, the polypropylene leaves were all plunged into a 1.8 ml cryovial filled with liquid nitrogen (Figure 2d). Cryovial was stored in liquid nitrogen for at least 1 week (Figure 2e).

For warming, polypropylene leaves loaded with seminiferous tubule were removed from liquid nitrogen and immediately inserted into ICSI dishes covered with mineral oil at 37℃ for 5–10 min (Figure 2f). The thawed testicular seminiferous tubules were put in a culture dish containing 100 μl fresh sperm medium (G‐GAMETE™) for washing; then, the washed seminiferous tubules were put in 25 μl sperm medium (G‐GAMETE™) droplets. Testicular seminiferous tubules were mechanically torn with two 1 ml syringe needles to make a testicular cell suspension and assess the motility rate, survival rate and DNA fragmentation rate of testicular spermatozoa.

#### 2.3.2 Testicular cell suspensions frozen with novel cryopiece

For freezing, the prepared 100 μl cryoprotector‐sperm mixture of G2 was divided into 5 parts, and a 20 μl droplet of cryoprotector‐sperm mixture on each of 5 non‐toxic polypropylene leaves was made. The method of cryopreservation of testicular cell suspensions using novel cryopiece was as above described.

For warming, polypropylene leaves loaded with sperm mixture were removed from liquid nitrogen and immediately inserted into ICSI dishes covered with mineral oil at 37℃ for 5–10 min. After warming, sperm mixture was transferred into 1ml of G‐GAMETE™ medium, centrifuged at 500 g for 10 min. Then, the supernatant was discarded, and the pellet was resuspended in 25 µl of sperm medium and kept at 37℃ for at least 1 h until it was assayed for sperm motility, survival and DFI.

#### 2.3.3 Testicular cell suspensions frozen with 0.25 ml straw (CryoBioSystem, Paris, France)

For freezing, the prepared 100 μl cryoprotector‐sperm mixture of G3 was aspirated into a 0.25 ml straw; then, the straw was covered with an obturation and identification rod, placed 4 cm above the surface of liquid nitrogen for 10 min and then stored in liquid nitrogen for at least 1 week before warming (Gianaroli et al., 1999).

For warming, the straw was warmed in the water bath for 1 min at 37℃, the obturation and identification rod were removed, and the cryoprotector‐sperm mixture was put in a centrifuge tube containing 1 ml of G‐GAMETE™ medium and centrifuged at 500 g for about 5 min. The supernatant was discarded, and the pellet was resuspended in 25 µl sperm medium and kept at 37℃ until it was assayed for sperm motility, survival and DFI.

### 2.4 Sperm quality assessment

The DNA fragmentation rate of sperm samples was determined by the sperm chromatin diffusion test using the Sperm DNA Fragmentation Staining Kit (BredLifeScience) (Zhou et al., 2016). Nuclei with large or medium size halos were considered spermatozoa with non‐fragmented DNA, whereas nuclei with small size halos or degradation were considered spermatozoa with fragmented DNA. At least 200 spermatozoa per sample were scored. Sperm motility and survival rate were evaluated according to the World Health Organization (WHO) criteria (Cooper et al., 2010).

### 2.5 Statistical analysis

Statistical analysis was carried out by using the software package. Data are expressed as mean ± standard deviation. T test for paired samples was used to compare the testicular sperm motility rate, survival rate and DNA fragmentation rate before and after freezing. The relationship between unsuccessful post‐thaw sperm retrieval and sperm freezing method was examined by using the Fisher exact test for ordinal variables. Significance was set at *p *< 0.05.

## 3 RESULTS

**FIGURE 3:**
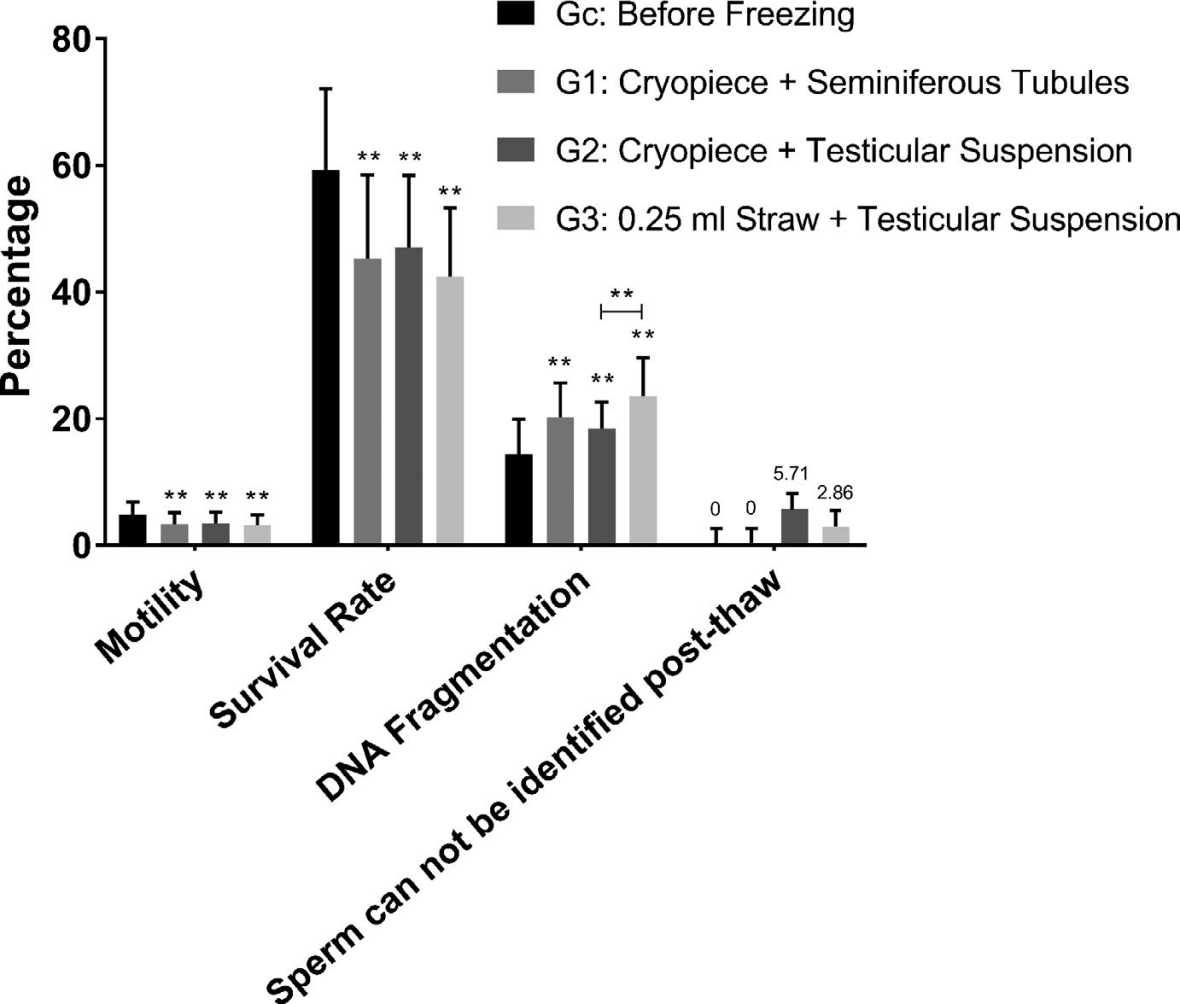
Testicular sperm parameters and retrieval rate in patients with obstructive azoospermia before and after cryopreservation with novel cryopiece, 0.25 mL straw

Human testicular tissue was obtained from 40 patients who underwent diagnostic testicular biopsy as a source of samples for cryopreservation test. The mean age of the patients was 31.48 years (range 22–51). A total of 35 patients suffered from OA, mostly as a result of congenital bilateral absence of the vas deferens (CBAVD) or seminal duct obstruction. 4 patients showed NOA resulting from chromosomal abnormalities, tumour chemoradiation and unexplained spermatogenic failure. 1 patient showed hypospermatogenesis due to advanced male age (Table 1). Excluding NOA and hypospermatogenesis testicular tissue samples, 35 OA testicular tissue samples were selected for cryopreservation tests. The motility rate, survival rate and DNA integrity of freeze–thaw testicular spermatozoa in G1, G2 and G3 experimental groups decreased to a variable extent compared with Gc in the control group (Figure 3).

Cryopreserved testicular spermatozoa by means of frozen testicular seminiferous tubules in G1 and frozen testicular tissue suspensions in G2 did not show significant differences in testicular sperm quality after thawing and resuscitation (Figure 3).

The motility and survival rates of freeze–thaw testicular sperm frozen with novel cryopiece in G2 were not different from those frozen with 0.25 ml straw in G3; however, the DFI was lower (20.27 ± 5.40 vs 23.55 ± 6.02; *p* = 0. 004) (Figure 3).

The freeze–thaw testicular spermatozoa could be found in all 35 testicular seminiferous tubule specimens in G1; however, it could not be found in 2 of 35 (5.7%) and 1 of 35 (2.9%) testicular cell suspensions samples in G2 and G3 respectively (Figure 3).

## 4 DISCUSSION

The small number of spermatozoa obtained by testicular biopsy is extremely precious for patients with obstructive azoospermia. The cryopreservation of human testicular spermatozoa has become an essential technique in assisted reproductive laboratories because it allows highly efficient use of testicular spermatozoa and avoids irreversible damage to the patient's testis caused by multiple testicular biopsies. However, how to cryopreserve testicular spermatozoa safely, effectively and economically has become a relevant research issue in this field. In this study, we developed a novel testicular sperm cryocarrier, named novel cryopiece, which presented for the cryopreservation of testicular seminiferous tubules and testicular cell suspension seems to be at least as effective as 0.25 ml straw cryocarrier. Our data show that the novel cryopiece is a homemade, simple and efficient cryocarrier with low cost of use and easy to be widely promoted in clinical practice.

To date, many containers have been designed for freezing a small number of spermatozoa, although they have been proven to have reasonable success; nevertheless, they have several drawbacks preventing their wide use in clinical practice. Initially, researchers used human or hamster empty zona pellucida as a cryocarrier for storing small numbers of spermatozoa. This method not only did avoid contamination from foreign heterogeneous protein contamination, but also brought a series of biological and ethical problems (Cohen et al., 1997; Ye et al., 2009). Researchers subsequently developed artificial zona pellucida carriers such as volvox globator algae (Just et al., 2004), agarosegel microspheres (Araki et al., 2015; Hatakeyama et al., 2017), as sperm freezing carriers, but because such carriers have not been used in clinical practice, their safety remains unclear. Some research groups tried to freeze single or small amounts of spermatozoa, using carriers designed for frozen eggs or frozen embryos, such as cryoloop (Nawroth et al., 2002) and cryotop (Chen et al., 2015). However, the storage system of cryoloop is not stable enough and requires a high technological level, while the Cryotop is an open storage system in which the frozen spermatozoon is in direct contact with liquid nitrogen; therefore, with this system, the risk of cross‐contamination cannot be avoided. In recent years, CellSleeper (Endo et al., 2012) and Cryopiece (Sun et al., 2017) have been used in clinical practice and have shown to reach high fertilization and live birth rates after ICSI of cryopreserved spermatozoa. However, these cryocarriers still have several shortcomings.

The novel cryopiece carrier we developed is suitable not only for the cryopreservation of sperm suspensions, but also for the cryopreservation of testicular seminiferous tubule. The cryocarrier consists only of non‐toxic polypropylene leaves, PVC foam tags and conventional sperm cryovials, which can be made by each reproductive centre easily.

In this paper, testicular cell suspensions were prepared by mechanical tissue depolymerization rather than enzymatic digestion. Compared with cryopreservation of testicular tissue, separation of single cell suspension requires enzymatic digestion, which might affect the viability of germ cells and change the biophysical characteristics of cells and increase the sensitivity of cells to the cryopreservation process. Evidence of cellular stress suggests a reduction in viability and functionality of cells in cell suspension after enzymatic digestion and resuspension (Brook et al., 2001; Griswold, 1998). Mechanical tissue depolymerization is an effective alternative to enzymatic digestion. High cell numbers, viability and spermatozoa enrichment have been reported following mechanical depolymerization of testicular tissue (Onofre et al., 2016).

The problem of contamination during sperm cryopreservation has been difficult to solve. While the risk of cross‐contamination during cryopreservation of spermatozoa and embryos is low, however, it still remains non‐negligible (Wu & Yao, 2010). Therefore, the search for safer freezing devices remains a worthwhile strategy to avoid the likelihood of cross‐contamination (Cobo et al., 2010). The cryotube with internal thread may provide better sealing, as shown in 1A, the novel cryopiece we designed encapsulates carrier leaves in a 1.8 ml cryotube to avoid direct contact between testicular tissue and liquid nitrogen and reduce the possibility of contamination of the specimen. Nevertheless, novel cryopiece cannot be considered as closed system, and the Nunc catalogues and the Cryopreservation Manual (Frank & Simione, 1998) recommend that cryotube submerged in liquid nitrogen must be properly sealed in NUNC™ CryoFlex™ tubing for secondary sealed in order to prevent cross‐contamination of spermatozoa.

An ideal cryocarrier would allow the freezing of multiple tiny aliquots of spermatozoa in small amounts (AbdelHafez et al., 2009). Novel cryopiece has multiple carrier leaves. Testicular seminiferous tubules can be divided into multiple portions and frozen on each carrier leaf. Cryopreserved seminiferous tubule on multiple carrier leaves can be used for multiple cycles of assisted reproduction, so allowing a substantial improvement in the utilization of testicular tissue. In addition, this study is the first to propose the attachment of foam tags to carrier leaves. As shown in groups 1 and 2, the foam tags can be floated on the liquid nitrogen, and the carrier leaf can be located and used according to the serial number on the foam tag, so enhancing the management of frozen spermatozoa.

In this study, we collected testicular tissue from 35 patients with obstructive azoospermia and compared the quality of testicular spermatozoa cryopreserved by different freezing methods. The motility rate, survival rate and DNA integrity of freeze–thaw testicular spermatozoa in G1, G2 and G3 experimental groups decreased to a variable extent compared with Gc in the control group. The freezing process has adverse effects on testicular spermatozoa due to the rupture of the plasma membrane caused by the formation of ice crystals in the cells during freezing (Dalzell et al., 2004; Verheyen et al., 1997). Our data showed that testicular spermatozoa frozen with novel cryopiece in G2 had a low sperm DFI compared with 0.25ml straw in G3; however, motility and survival rates were not significantly different. This is probably because novel cryopiece has a shorter cooling time of the only 5min compared with 0.25mL straw. Moreover, the time for complete melting of the cryoprotector‐sperm mixture was 2s in novel cryopiece versus 5s in 0.25 ml straw, and the thawing rate of novel cryopiece was faster than that of 0.25 ml straw (Wang et al., 2018). Uniform and rapid thawing times contribute to the formation of fewer ice crystals with reduced cell damage (Mazur, 1984).

The freeze–thaw testicular spermatozoa could be found in all 35 testicular seminiferous tubule specimens in G1; however, it could not be found in 2 of 35 (5.7%) and 1 of 35 (2.9%) testicular cell suspensions samples in G2 and G3 respectively.

In our study, spermatozoa could not be detected after thawing in 2 (5.7%) and 1 (2.9%) of 35 testicular cell suspensions samples in G2 and G3 respectively; this finding is in accordance with other studies (Chen et al., 2015; Donnelly et al., 2001). Conversely, spermatozoa could be found in all testicular seminiferous tubules after thawing in 35 testicular seminiferous tubule in G1. Since the seminiferous tubule wall remains intact, the method of freezing seminiferous tubule to cryopreserve testicular spermatozoa does not lead to the adhesion of spermatozoa to the carrier surface and the culture tube wall. Although failure to retrieve any spermatozoa after thawing testicular cell suspension is an infrequent event (Kathrins et al., 2017), cryopreservation of testicular spermatozoa by freezing the seminiferous tubule is a better option because it may lead to a higher sperm recovery rate.

The present study has several limitations, and it confirms the advantages of using novel cryopiece to freeze testicular seminiferous tubule to cryopreserve testicular spermatozoa. However, there is a lack of data on assisted reproductive outcomes to fully demonstrate the feasibility of this freezing method. Moreover, there are no data on the effects of novel cryopiece on embryos. Our next research will investigate this issue, in order to apply novel cryopiece to clinical practice as soon as possible. In conclusion, novel cryopiece is easy to be performed and can freeze multiple tiny aliquots of spermatozoa in small amounts, and can also freeze testicular seminiferous tubule, improving testicular sperm utilization. Cryopreservation of testicular spermatozoa with novel cryopiece, especially in patients with obstructive azoospermia, is simple and effective and should be widely used.

## CONFLICTS OF INTEREST

No conflicts of interest.

## Data Availability

The data that support the findings of this study are available on request from the corresponding author. The data are not publicly available due to privacy or ethical restrictions.
